# Correlation between Apelin and Some Angiogenic Factors in the Pathogenesis of Preeclampsia: Apelin-13 as Novel Drug for Treating Preeclampsia and Its Physiological Effects on Placenta

**DOI:** 10.1155/2021/5017362

**Published:** 2021-11-15

**Authors:** Reham Z. Hamza, Abdel Aziz A. Diab, Mansour H. Zahra, Ali K. Asalah, Suzan M. M. Moursi, Najah M. Al-Baqami, Fawziah A. Al-Salmi, Mai S. Attia

**Affiliations:** ^1^Biology Department, Faculty of Sciences, Taif University, P.O. Box 11099, Taif 21944, Saudi Arabia; ^2^Zoology Department, Faculty of Science, Zagazig University, Zagazig 44519, Egypt; ^3^Medical Physiology Department, Faculty of Human Medicine, Zagazig University, Zagazig 44519, Egypt; ^4^Department of Biological Sciences, Zoology, King Abdulaziz University, Jeddah 21589, Saudi Arabia

## Abstract

Preeclampsia (PE) is one of the commonest causes for maternal and fetal morbidity and mortality. Imbalances of angiogenic factors, oxidative stress, and inflammatory response have a role in the pathogenesis of PE. Data regarding the circulating apelin level and its role in PE remains controversial. This study was formulated to assess the serum apelin level in PE, investigate its correlation with some inflammatory, oxidative stress, and angiogenic proteins in a nitric oxide synthase inhibitor; the N (gamma)-nitro-L-arginine methyl ester (L-NAME)-induced rat model of PE and determine whether apelin administration could protect against development of PE. 40 healthy adult female albino rats and 10 adult male albino rats were used in this study. The pregnant female rats were randomly divided into three groups: group 1 (normal pregnant group), group 2 (PE-induced group), injected subcutaneously with 75 mg L-NAME/kg bodyweight/day starting from day 9 to 20 of gestation, and group 3 (PE-induced group supplemented with apelin (PE + apelin)); PE induced as before and simultaneously subcutaneously injected with apelin-13 (6 × 10^−8^ mol/kg bodyweight/twice daily) beginning from day 6 to 20 of gestation. In all groups, blood pressure and urine protein were determined at gestation days (GD) 0, 10, and 18. Moreover, serum apelin, placental growth factor (PLGF), vascular endothelial growth factor (VEGF), soluble fms-like tyrosine kinase-1 (sFlt-1), soluble endoglin (sEng), interferon-gamma (IFN-*γ*), and interleukin-10 (IL-10) levels and serum superoxide dismutase enzyme (SOD) and catalase (CAT) activities of all groups were estimated at the end of experiment. Placental histopathological examination was also performed. PE-induced rats showed significantly decreased serum apelin levels. Moreover, they showed significantly increased blood pressures, urine proteins, sFlt-1, sEng, and IFN-*γ* (mean arterial blood pressure, urine proteins, sFlt-1, sEng, and IFN-*γ* showed significant negative correlations with serum apelin level), but it showed significantly decreased VEGF, PLGF, IL-10, SOD, and CAT (VEGF, PLGF, IL-10, and SOD showed significant positive correlations with serum apelin level). In contrast, exogenous apelin administration significantly ameliorated these parameters together with improvement in the placental histoarchitecture in the apelin-supplemented PE group. This study demonstrated the protective effects of apelin administration on the pathogenesis of PE.

## 1. Introduction

Preeclampsia (PE) is characterized by a newly developed arterial hypertension associated with one of these complications: proteinuria, maternal organ injury, and uteroplacental dysfunction [1]. It is one of the primary reasons of intrauterine growth retardation, high maternal morbidity, and premature birth [2].

The exact mechanism of pathophysiology of preeclampsia remains unknown. However, abnormal placentation, angiogenic factors levels disproportion, increased inflammation, and oxidative stress in the placenta play an important role in the pathogenesis of this syndrome [3]. In preeclampsia, the essential trophoblasts transformation from epithelial phenotype to endothelial phenotype that involves direct contact with maternal blood is incomplete. Cytotrophoblasts invade to superficial decidual parts of placenta only and do not reach myometrial parts which consequently lead to placental insufficiency that triggers tissue oxidative stress, apoptosis, necrosis of placental tissue, an exaggerated inflammatory response, endothelial dysfunction, and finally intrauterine growth restriction and intrauterine death [4].

Moreover, preeclampsia diagnosis remains disputing because it depends on nonspecific markers such as proteinuria and hypertension that are not useful when the pregnant women have preexistent hypertension or proteinuria like in case of renal diseases [5].

Treatment options also remain limited yet, and discovery of a safe drug that can decrease blood pressure and mitigate disease progression is of major concern [6].

Several adipocytokines including resistin, adiponectin, and apelin are released from placenta beside the adipose tissues during pregnancy [7]. Apelin is a small regulatory peptide, and it is the endogenous ligand of the orphan G protein coupled receptor, apelin receptor (APJ). It has various isoforms whose effects change according to the forms. 13 and 17 amino acids isoforms are biologically stronger than its form consisting of 36 amino acids [8].

Studies on the correlation between the circulating apelin level and PE yielded inconsistent findings and remained controversial. Although several studies demonstrated a downregulated apelin/APJ system in preeclampsia [9–11], others found increased apelin maternal levels and placenta expression in preeclampsia compared to healthy women [12, 13].

On another note, it has been demonstrated that apelin can decrease the arterial blood pressure in several animal models and heart failure patients [14, 15]. It is also necessary for normal vascular development, and its role in both normal and pathologic angiogenesis has been suggested. As apelin has been involved in blood pressure regulation, angiogenesis, and fluid balance, apelin and its receptor (APJ) might play a significant role in the pathophysiology of preeclampsia [16].

Therefore, this study aimed to determine apelin circulating level and its relation to some angiogenic factors, antioxidant enzymes, and inflammatory markers and investigate the effects of apelin supplementation on the L-NAME-induced rat model of PE.

## 2. Materials and Methods

This study was conducted in Faculty of Medicine, Zagazig University, and used 50 healthy albino rats (40 virgin females (119–140 g, 50 days old) and 10 adult males used for fertilization (210–238 g)) attained from Faculty of Veterinary Medicine, Zagazig University. Rats were retained in wired cages in a clean and sanitary conditions and were fed the normal commercial rodent chow with free water supplementation and stayed on a 12 h light/dark cycle at room temperature. All the experiments have been conducted in agreement with the guideline for the care and use of research animals reviewed and approved by the ZU-IACUC Committee, approvingly number ZU-IACUC/1/F/78/2019.

### 2.1. Pregnancy Evocation

Female rats were examined for estrous cycles for 2 consecutive weeks. Vaginal smears were examined microscopically every morning, and the phases of the estrus cycle were determined through investigation of the vaginal cytological examination. The estrous phase was detected by increasing the number of cornified epithelia cells that finally predominate as the estrus progresses.

Rats in the estrous phase were left to mate with a mature male rat in a separate cage and then examined for copulation in the next morning by the appearance of a copulation plug or sperms in the vaginal smears. The presence of sperms denotes the first gestational day [17]. 7 rats did not conceive and so were ruled out from the study.

Thirty-three pregnant rats were randomly assembled into three groups: group 1 (normal pregnant control group, *n* = 10) was injected by subcutaneous (s.c) saline from day 9 to 20 of gestation. Group 2 (preeclampsia induced group, *n* = 12) was injected subcutaneously with 75 mg/kg bodyweight/day L-NAME (total volume 9–10.5 mg daily/rat) (Sigma Aldrich Co., USA) starting from day 9 to 20 of gestation [18]. Group 3 (preeclampsia-induced group treated with apelin, *n* = 11) gave L-NAME at the same dose and timing of group 2 and simultaneously injected subcutaneously with 6 × 10^−8^ mol/kg/twice daily apelin-13 (total volume 1.2 × 10^−2^ mg/twice daily/rat) (Sigma Aldrich Co., USA) starting from day 6 to 20 of pregnancy [14], whereas the other two groups were given saline instead (This chosen dose of apelin-13 ameliorates the pathologies of preeclampsia induced by reduced uterine perfusion pressure, no rats died, and the statistics were performed by using 10 rats from each group).

### 2.2. Measurement of Blood Pressure (BP)

On days 0, 10, and 18 of gestation, systolic, diastolic, and mean arterial blood pressures were measured using the noninvasive BP measurement system (NIBP250/serial no. 21202–108/BIOPAC system, Inc., USA) [19].

### 2.3. Urine Collection

Metabolic cages were used for collecting 24 hours urine samples on days 0, 10, and 18 of pregnancy. The urine samples were centrifuged 10 minutes at approximately 3000 rpm to remove insoluble materials. The supernatants were kept in other tubes and stored at –20°C till used.

### 2.4. Measurement of Urine Total Proteins

Urine Total Proteins measurement was carried out as described by Nishi and Elin [20] using the urinary protein assay kit (Chondrex Inc., 2607–151 place NE, Redmond, WA 98052, USA).

### 2.5. Blood Sampling

Blood samples from retroorbital veins were obtained at the ending of the 20th day of gestation and then allowed to clot and centrifuged at 3000 rpm for 20 minutes to separate serum that has been kept at −20°C until used for analysis.

### 2.6. Serum Analysis

#### 2.6.1. Estimation of Serum Apelin

Serum apelin was estimated following manufacturer's instructions using rat apelin Elisa kit (Shanghai Crystal Day Biotech Co. (SCDB Co.), Ltd., catalog number E0813Ra).

#### 2.6.2. Estimation of Serum VEGF

Serum VEGF was measured using rat VEGF Elisa kit (SCDB Co., Ltd., catalog number E0659Ra) following their manufacturer's recommendations.

#### 2.6.3. Estimation of Serum PLGF

Serum PLGF was measured using rat PLGF Elisa kit (SCDB Co., Ltd., catalog number E0579Ra) following their manufacturer's recommendations.

#### 2.6.4. Estimation of Serum sFlt-1

Serum sFlt-1 was estimated using rat sFlt-1 Elisa kit (SCDB Co., Ltd., catalog number E1233Ra) following their manufacturer's recommendations.

#### 2.6.5. Estimation of Serum sEng

Serum sEng was measured using rat sEng Elisa kit (Bioassay Technology Laboratory (BT Lab), catalog number E1557Ra) following their manufacturer's recommendations.

#### 2.6.6. Estimation of Serum SOD Activity

Serum SOD activity was estimated as described by [21] using Elisa kit (Biodiagnostic Company, Dokki, Giza, Egypt).

#### 2.6.7. Estimation of Serum CAT Activity

Serum CAT activity was estimated as described by [22] using Elisa kit (Biodiagnostic Company, Dokki, Giza, Egypt).

#### 2.6.8. Estimation of Serum IL-10 and Serum IFN-*γ*

Serum IL-10 and serum IFN-*γ* were measured following their manufacturer's instructions using specific rat Elisa kits from RayBiotech.com.

### 2.7. Histopathological Examination

Placentas of all groups were removed and placed in 10% formalin for 24 hours. Then, placental tissues were fixed in paraffin. 3–5 *μ*m thick serial sections were cut from the tissue embedded paraffin blocks, placed on slides, and stained with hematoxylin and eosin (H&E) for studying the general microscopic characteristics.

### 2.8. Statistical Analysis

Data were introduced as mean ± SD and analyzed statistically via SPSS version 19 (SPSS Inc/Chicago/United States). Analysis of variance (ANOVA) and the LSD post hoc test were performed to compare between different groups. Pearson's correlation analysis was conducted to identify the correlations between parameters. *P* values less than 0.05 were considered statistically significant for all tests performed.

## 3. Results

**Figure 1:**
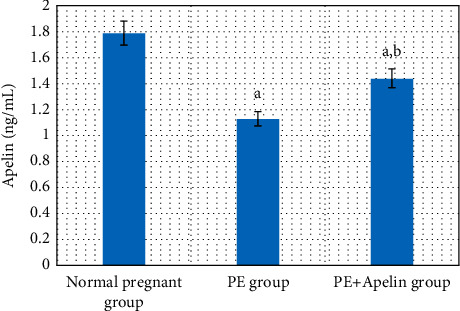
Serum apelin concentration in the studied groups.

**Figure 2:**
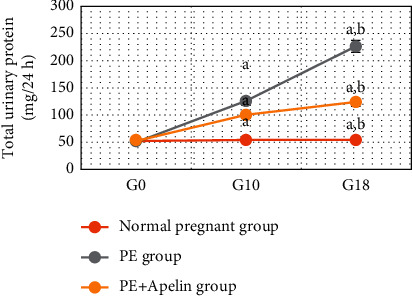
Urine proteins in the studied groups.

**Figure 3:**
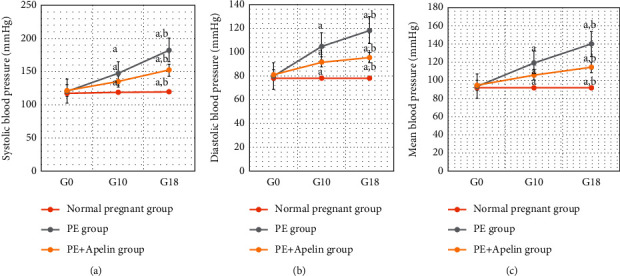
(a)–(c) Systolic, diastolic, and mean arterial blood pressures in all studied groups.

**Figure 4:**
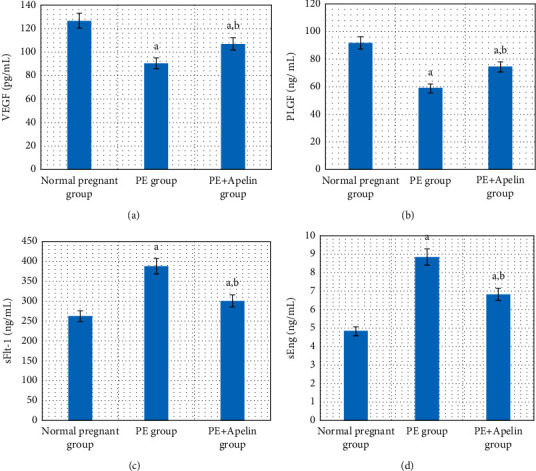
(a)–(d) Serum VEGF, PLGF, sFlt-1, and sEng in all studied groups.

**Figure 5:**
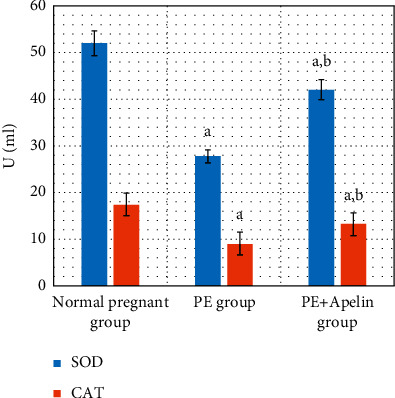
Serum SOD and CAT activities in all studied groups.

**Figure 6:**
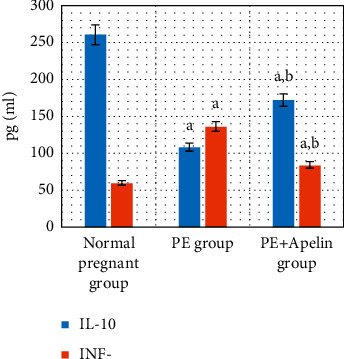
Serum IL-10 and IFN-*γ* in all studied groups.

In the PE group, rats exhibited significant decreases in serum apelin, VEGF, PLGF, IL-10 levels, and SOD and CAT activities (*P* < 0.001) in comparison to the normal pregnant group. In addition, increased arterial blood pressures, urine protein, serum sFlt-1, sEng, and IFN-*γ* (*P* < 0.001) were also detected in the PE group in comparison to normal pregnant one (Figures 1–6).

On the other hand, it was found that apelin supplementation significantly decreased arterial blood pressure, urinary protein, serum sFlt-1, sEng, and IFN-*γ* (*P* < 0.001), but it significantly elevated serum VEGF, PLGF, IL-10 levels, and SOD and CAT activities (*P* < 0.001) in the PE group supplemented with apelin (Figures 1–6).

Furthermore, the serum apelin level showed significant positive correlations with serum VEGF, PLGF, and IL-10 levels and SOD activity but showed significant negative correlations with mean arterial blood pressure, serum sFlt-1, IFN-*γ*, and urine protein (Figures 7–14).

## 4. Discussion

**Figure 15:**
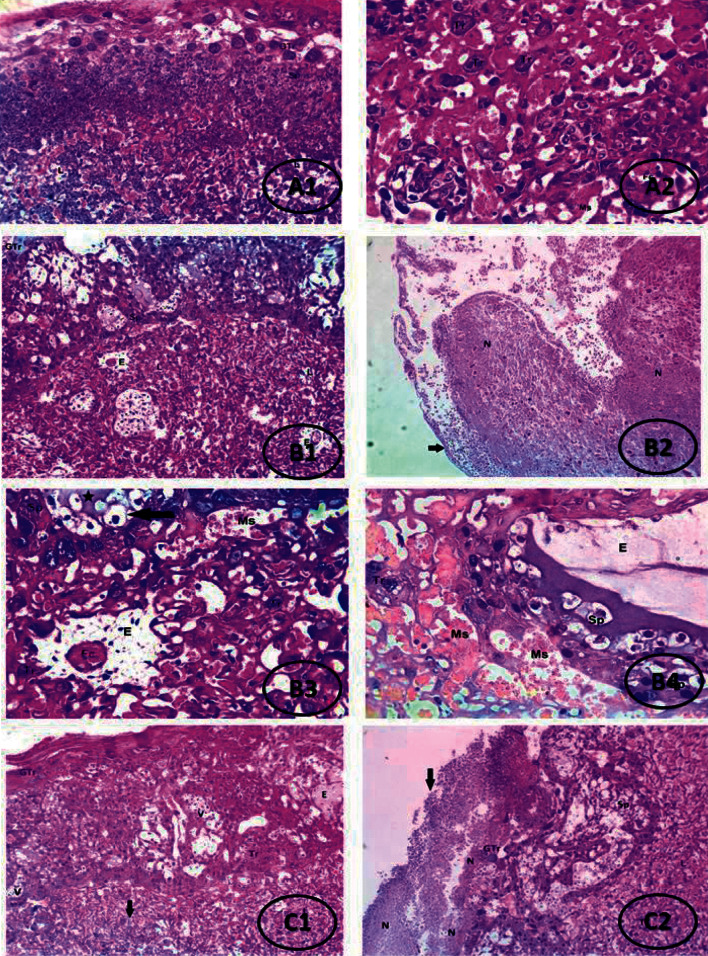
Photomicrographs of placenta of control and different treated groups. (A1, A2) Photomicrograph of placenta of the normal pregnant group showing normal placental histology. Giant trophoblasts (GTr) and spongioblasts (Sp) showing normal labyrinth histology. Tr, trophoblasts, Fc, fetal capillaries, Ms, maternal sinuses (Ms), and L, labyrinth (H&E, X100). (B1–B4) Photomicrograph of placenta of the PE group showing edema (E) and fluid accumulation in the interstitial space between spongioblasts. GTr, giant trophoblasts, Sp, spongioblasts, L, labyrinth, and N, wide areas of necrosis, including the whole placenta leaving only ghosts of cells with infiltration of inflammatory cells (black arrow) (H&E, X100), and edema (E) in the interstitial space between spongioblasts (asterisk) with hydropic degeneration in some spongioblasts (black arrow). In the labyrinth, foci of villous edema surrounding the fetal capillaries are seen. Fetal capillaries show thrombus formation. Tr, trophoblasts, Fc, fetal capillaries, Ms, maternal sinuses, Sp, spongioblasts (H&E, X400). (C1, C2) Photomicrograph of placenta of the apelin-treated PE group showing mild placental changes including degenerative changes in some giant trophoblastic cells, vacuolar degeneration in some spongiotrophoblast (V), and mild inflammatory reaction (black arrow) in the labyrinth (L) (H&E, X100).

Preeclampsia is a determined pregnancy problem as it affects 3–8% of pregnancies, negatively affects both the mother and fetus, and increases the risk of all-causing mortality [23]. Its symptoms are well-defined, but the pathophysiology is not fully understood, and there is no therapy available for reversing, preventing, stabilizing, or curing the disease [24]. Thus, an effective treatment to prevent and treat preeclampsia is required, and apelin might be a novel potential agent that helps in diagnosis and treatment of preeclampsia [14] (Figure 15).

Apelin has several cardiac and vascular effects including angiogenesis, arterial blood pressure regulation, cell differentiation, and fluid balance [25]. Moreover, apelin expression is observed in a wide range of peripheral tissues, as cardiac, hepatic, renal, and adipose tissues with high level of expression in the lung, mammary glands, and placenta [26].

Thus, we aimed in this study to assess the correlation between serum apelin and some inflammatory, oxidative stress, and angiogenic proteins in the L-NAME-induced rat model of PE and to evaluate its possible protecting effect against development of PE symptoms.

The results of our study showed a significant decrease in apelin circulating level in the PE group compared to the normal pregnant group. This agrees with the results of Deniz et al. [27] and Gürlek et al. [28], but contradicted by other studies which detect higher apelin circulating levels in preeclampsia [9, 13]. That may be because different studies have investigated different isoforms of apelin [28]. Than et al. [29] also stated that the apelin level is different during pregnancy since apelin is first excreted from adipocytes, and imbalances in the adipose tissue volume might be another reason for varying apelin levels among research studies.

Circulating apelin level decreased in the middle of pregnancy, but it tends to rise in the third trimester in healthy pregnancy [30]. As higher apelin concentrations are expected in the last trimester of normal pregnancy, lower maternal concentrations may play a key role in the etiology of preeclampsia that is primarily characterized by pathological angiogenesis [28].

Moreover, apelin circulating levels showed significant negative correlations with mean arterial blood pressure and with proteinuria degree in the PE group in our study. However, apelin provision in preeclamptic rats (PE + apelin) significantly decreased the elevated systolic, diastolic, and mean arterial blood pressures and reversed the PE-associated proteinuria indicating amelioration of preeclampsia symptoms. Our results agree with other findings showing lowering blood pressure effects, normalized proteinuria, and improved renal pathology by pyroglutamate apelin-13 (Pyr-apelin-13) [31].

Apelin could elevate the level of nitric oxide (NO) or its bioavailability and so can ameliorate endothelial cells dysfunction and decrease vascular resistance [32]. Apelin has a direct activating effect on the L-arginine endothelial nitric oxide synthase (eNOS)/nitric oxide pathway [33].

It is hypothesized that abnormal placentation and an imbalance in the expression of angiogenic, including vascular endothelial growth factor and placenta growth factor, and antiangiogenic factors, including soluble fms-like tyrosine kinase-1 (sFlt-1) and soluble endoglin (sEng), appear to be major contributors [34]. Immune maladaptation associated with initial insufficient invasion of trophoblast and abnormal uterine spiral artery remodeling inducing uteroplacental vascular insufficiency and ischemia leads to release of placental factors such as reactive oxygen species and proinflammatory cytokines into maternal circulation that can cause a massive inflammatory cascade, which is another important step in the pathogenesis of preeclampsia [1].

Multiple studies have attempted to update the definition of PE by incorporation of crucial biomarkers released from either placenta or blood vessels, such as VEGF, PlGF, sFlt-1, or sEng in the diagnosis of preeclampsia and even predicting disease development and its outcome [35–37].

In our study, serum PLGF and VEGF levels were decreased with significant positive correlation with serum apelin in the PE group compared to the control group. The PE group also showed significantly increased serum sFlt-1 and sEng levels when compared to the normal pregnant group. However, apelin administration significantly improved their values in the treated group. These results point out the ameliorating effect of apelin in the pathogenesis of PE.

Normal placentas release VEGF and PlGF. Both are crucial for normal vascular development and activate both vascular endothelial growth factor receptor-1 (VEGFR-1) and vascular endothelial growth factor receptor-2 (VEGFR-2) linked to eNOS that is required for normal angiogenesis, which allow the placenta to build an adequate vascular network for the fetus's development [38]. A soluble form of VEGF or PlGF receptors (sFlt-1) increases in the third trimester and floats freely in the placenta and maternal serum. It is believed that excess sFlt-1 can cause preeclampsia [39].

sFlt-1 is induced by hypoxia inducible factor-1 and acts as an effective scavenger of VEGF and PlGF and therefore induced vascular endothelium dysfunction [40]. sFlt-1 also sensitizes the maternal vascular endothelium to proinflammatory cytokines as tumor necrosis factor-*α* (TNF-*α*) producing generalized endothelium dysfunction and multiorgan damage [41].

Soluble endoglin (sENG) is a glycoprotein generated by proteolytic splitting of the extracellular portion of endoglin (the transmembrane coreceptor for transforming growth factor-beta (TGF-*β*) receptor) that acts as a limiting factor for TGF-*β* and the associated eNOS activity [42]. As TGF-*β* has anti-inflammatory, vasodilator, and growth effects, its action blockage by sEng results in endothelial dysfunctions characterized by vasoconstriction and overexpression of adhesion molecules designating preeclampsia [43].

The binding of apelin to APJ/phosphatidylinositol-3 kinase (PI3K)/extracellular signal regulated kinase (ERK) pathway induces endothelial cell migration and proliferation [44]. Apelin can mediate angiogenesis via upregulation of VEGF or VEGF receptor-2 [45]. When the angiogenic and vasodilatation effects of apelin are considered, increasing apelin circulating levels in preeclampsia patients may affect mother and fetus outcomes positively by improving the maternal adaptation to pregnancy [28].

Furthermore, oxidative stress has an important role in preeclampsia progress. It can trigger apoptosis of syncytium and consequently increase proinflammatory cytokines and antiangiogenic factors secretions, which eventually induced preeclampsia [46]. Several studies have showed that preeclampsia is accompanied with high oxidative stress biomarkers levels, involving lipid peroxides and decreased antioxidant magnitude [6, 47]. In line with these findings, the present study also demonstrated significantly decreased serum SOD (positively correlated with serum apelin level) and CAT activities in the PE group.

Moreover, significantly decreased serum IL-10 that correlated positively with serum apelin combined with significant increases in IFN-*γ* serum levels that correlated negatively with serum apelin have been found in the PE group. These results are in line with Murthi et al. [48] and Armistead et al. [49] who stated that the inflammation is one of the mechanisms of preeclampsia as the imbalance between levels of IL-10 and IFN-*γ* inhibits trophoblastic invasion that encourages placental ischemia and induces endothelial dysfunction.

On the other hand, our study demonstrated improved antioxidant enzyme activities (SOD and CAT) and inflammatory cytokines levels (IL-10 and IFN-*γ*) by apelin administration in PE rats. Apelin has also been found to suppress oxidative stress in various cell lines and tissue types [14, 50]. Apelin can also inhibit inflammatory responses by decreasing the generation of proinflammatory cytokines as monocyte chemoattractant protein-1, macrophage inflammatory protein-1*α*, TNF-*α*, and interleukin-1*β* and increasing the antiapoptotic cytokine IL-10 via stimulating the AMP-activated protein kinase (AMPK)/glycogen synthase kinase 3*β* (GSK-3*β*)/nuclear factor erythroid 2-related factor 2 (Nrf2) pathways [51, 52].

In addition, histopathological examination of the preeclamptic placenta by light microscopy revealed abnormal placental histology such as hydropic degeneration in some spongioblasts and edema and fluid accumulation in the interstitial spaces between spongioblasts with thrombi formation in fetal capillaries and villous edema surrounding it in the labyrinth. These findings complied with those of Raafat and Fathy [53], Tal et al. [54], and Hatice et al. [55]. Noteworthy, apelin administration improved the PE-associated changes in the placentas of the treated preeclamptic group.

In conclusion, our study demonstrated that apelin ameliorated the pathogenesis of preeclampsia. Restoring angiogenic/antiangiogenic balance, improving antioxidant status, and inhibiting inflammation may be engaged in the beneficial effects of apelin in preeclampsia. Apelin is a potential therapy for prevention and treatment of preeclampsia.

## Figures and Tables

**Figure 7:**
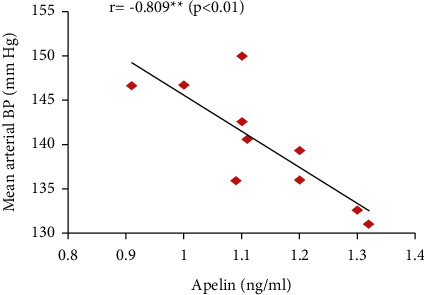
Correlation between serum apelin and mean arterial BP in the PE group.

**Figure 8:**
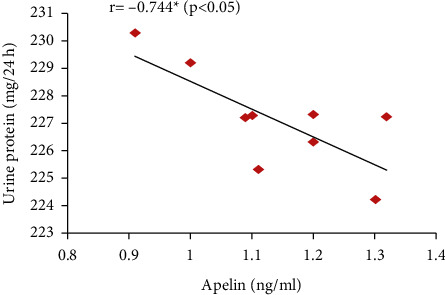
Correlation between serum apelin level and total urine proteins in the PE group.

**Figure 9:**
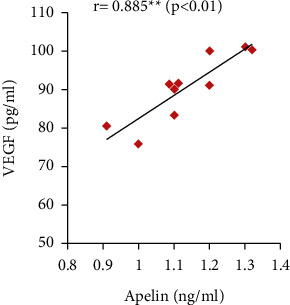
Correlation between serum apelin and VEGF in the PE group.

**Figure 10:**
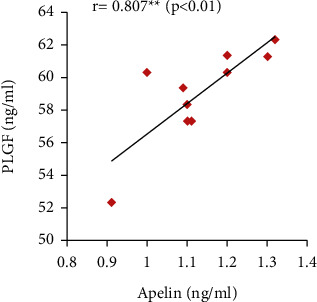
Correlation between serum apelin and PLGF in the PE group.

**Figure 11:**
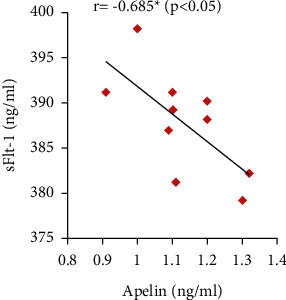
Correlation between serum apelin and sFlt-1 in the PE group.

**Figure 12:**
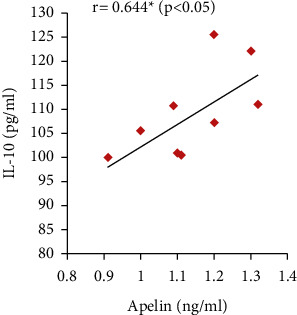
Correlation between serum apelin and IL-10 in the PE group.

**Figure 13:**
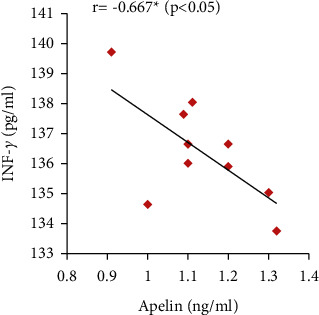
Correlation between serum apelin and IFN-*γ* in the PE group.

**Figure 14:**
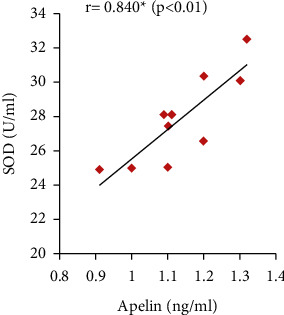
Correlation between serum apelin and SOD activity in the PE group.

## Data Availability

The data used to support the findings of this study are available from the corresponding author upon request.

## Additional Points

Increasing number of pregnant rats in the study needs to be conducted.

## Supplementary Materials

Supplementary Materials Attached supplementary files show measurement of the blood pressure of animals and anatomical features of pregnant rats.

## References

[1] Mayrink J., Costa M. L., Cecatti J. G. (2018). Preeclampsia in 2018: revisiting concepts, physiopathology, and prediction. *The Science World Journal*.

[2] Farhood T. H., Ewadh M. J., Alshaik S. F. (2020). Reduced glutathione, lipid peroxidation and malondialdehyde status in women with mild and severe preeclampsia for babylon governorate. *Indian Journal of Forensic Medicine & Toxicology*.

[3] Staff A. C. (2019). The two-stage placental model of preeclampsia: an update. *Journal of Reproductive Immunology*.

[4] Daskalakis G., Papapanagiotou A. (2015). Serum markers for the prediction of preeclampsia. *Journal of Neurology & Neurophysiology*.

[5] Hagmann H., Thadhani R., Benzing T., Karumanchi S. A., Stepan H. (2012). The promise of angiogenic markers for the early diagnosis and prediction of preeclampsia. *Clinical Chemistry*.

[6] Li Q., Yin L., Si Y., Zhang C., Meng Y., Yang W. (2020). The bioflavonoid quercetin improves pathophysiology in a rat model of preeclampsia. *Biomedicine & Pharmacotherapy*.

[7] Cobellis L., De Falco M., Mastrogiacomo A. (2007). Modulation of apelin and APJ receptor in normal and preeclampsia-complicated placentas. *Histology and Histopathology*.

[8] Beltowski J. (2006). Apelin and visfatin: unique “beneficial” adipokines upregulated in obesity?. *Medical Science Monitor*.

[9] Inuzuka H., Nishizawa H., Inagaki A. (2013). Decreased expression of apelin in placentas from severe pre-eclampsia patients. *Hypertension in Pregnancy*.

[10] Sattar Taha A., Zahraei Z., Al-Hakeim H. K. (2020). Serum apelin and galectin-3 in preeclampsia in Iraq. *Hypertension in Pregnancy*.

[11] Yamaleyeva L. M., Chappell M. C., Brosnihan K. B. (2015). Downregulation of apelin in the human placental chorionic villi from preeclamptic pregnancies. *American Journal of Physiology - Endocrinology And Metabolism*.

[12] Bortoff K. D., Qiu C., Runyon S., Williams M. A., Maitra R. (2012). Decreased maternal plasma apelin concentrations in preeclampsia. *Hypertension in Pregnancy*.

[13] Kucur M., Tuten A., Oncul M. (2014). Maternal serum apelin and YKL-40 levels in early and late-onset pre-eclampsia. *Hypertension in Pregnancy*.

[14] Wang C., Liu X., Kong D. (2017). Apelin as a novel drug for treating preeclampsia. *Experimental and Therapeutic Medicine*.

[15] Wu D., He L., Chen L. (2014). Apelin/APJ system: a promising therapy target for hypertension. *Molecular Biology Reports*.

[16] Estienne A., Bongrani A., Reverchon M. (2019). Involvement of novel adipokines, chemerin, visfatin, resistin and apelin in reproductive functions in normal and pathological conditions in humans and animal models. *International Journal of Molecular Sciences*.

[17] Abdul Aziz S. H., John C. M., Mohamed Yusof N. I. S. (2016). Animal model of gestational diabetes mellitus with pathophysiological resemblance to the human condition induced by multiple factors (nutritional, pharmacological, and stress) in rats. *BioMed Research International*.

[18] Shu W. E. N., Li H., Gong H. (2018). Evaluation of blood vessel injury, oxidative stress and circulating inflammatory factors in an L-NAME-induced preeclampsia-like rat model. *Experimental and therapeutic medicine*.

[19] Abubakar M. G., Ukwuani A. N., Mande U. U. (2015). Antihypertensive activity of *Hibiscus sabdariffa* aqueous calyx extract in Albino rats. *Sky Journal of Biochemistry Research*.

[20] Nishi H. H., Elin R. J. (1985). Three turbidimetric methods for determining total protein compared. *Clinical Chemistry*.

[21] Nishikimi M., Rao N. A., Yagi K. (1972). The occurrence of superoxide anion in the reaction of reduced phenazine methosulfate and molecular oxygen. *Biochemical and Biophysical Research Communications*.

[22] Aebi H. (1984). [13] Catalase in vitro. *Methods in Enzymology*.

[23] Jim B., Karumanchi S. A. (2017). Preeclampsia: pathogenesis, prevention, and long-term complications. *Seminars in Nephrology*.

[24] Weinreb H. (2018). Possible causes of preeclampsia and potential treatments. *The Science Journal of the Lander College of Arts and Sciences*.

[25] Kleinz M. J., Davenport A. P. (2005). Emerging roles of apelin in biology and medicine. *Pharmacology & Therapeutics*.

[26] Roberts E. M., Pope G. R., Newson M. J. F., Landgraf R., Lolait S. J., O’Carroll A. M. (2010). Stimulus‐specific neuroendocrine responses to osmotic challenges in apelin receptor knockout mice. *Journal of Neuroendocrinology*.

[27] Deniz R., Baykus Y., Ustebay S., Ugur K., Yavuzkir Ş., Aydin S. (2019). Evaluation of elabela, apelin and nitric oxide findings in maternal blood of normal pregnant women, pregnant women with pre-eclampsia, severe pre-eclampsia and umbilical arteries and venules of newborns. *Journal of Obstetrics and Gynaecology*.

[28] Gürlek B., Yılmaz A., Durakoğlugil M. E. (2020). Evaluation of serum apelin‐13 and apelin‐36 concentrations in preeclamptic pregnancies. *Journal of Obstetrics and Gynaecology Research*.

[29] Than A., Tee W. T., Chen P. (2012). Apelin secretion and expression of apelin receptors in 3T3-L1 adipocytes are differentially regulated by angiotensin type 1 and type 2 receptors. *Molecular and Cellular Endocrinology*.

[30] Van Mieghem T., Doherty A., Baczyk D., Drewlo S., Baud D., Carvalho J. (2016). Apelin in normal pregnancy and pregnancies complicated by placental insufficiency. *Reproductive Sciences*.

[31] Yamaleyeva L. M., Brosnihan K. B., Elsangeedy E. (2019). Systemic outcomes of (pyr 1)-apelin-13 infusion at mid-late pregnancy in a rat model with preeclamptic features. *Scientific Reports*.

[32] Azizi Y., Faghihi M., Imani A., Roghani M., Nazari A. (2013). Post-infarct treatment with [Pyr1]-apelin-13 reduces myocardial damage through reduction of oxidative injury and nitric oxide enhancement in the rat model of myocardial infarction. *Peptides*.

[33] Busch R., Strohbach A., Pennewitz M. (2015). Regulation of the endothelial apelin/APJ system by hemodynamic fluid flow. *Cellular Signalling*.

[34] Howell K. R., Powell T. L. (2017). Effects of maternal obesity on placental function and fetal development. *Reproduction (Cambridge, England)*.

[35] Baltajian K., Bajracharya S., Salahuddin S. (2016). Sequential plasma angiogenic factors levels in women with suspected preeclampsia. *American Journal of Obstetrics and Gynecology*.

[36] Palomaki G. E., Haddow J. E., Haddow H. R. (2015). Modeling risk for severe adverse outcomes using angiogenic factor measurements in women with suspected preterm preeclampsia. *Prenatal Diagnosis*.

[37] Sircar M., Thadhani R., Karumanchi S. A. (2015). Pathogenesis of preeclampsia. *Current Opinion in Nephrology and Hypertension*.

[38] Veron D., Villegas G., Aggarwal P. K. (2012). Acute podocyte vascular endothelial growth factor (VEGF-A) knockdown disrupts alphaVbeta3 integrin signaling in the glomerulus. *PLoS One*.

[39] Redman C. W., Sargent I. L. (2005). Latest advances in understanding preeclampsia. *Science*.

[40] Onda K., Tong S., Beard S. (2017). Proton pump inhibitors decrease soluble fms-like tyrosine kinase-1 and soluble endoglin secretion, decrease hypertension, and rescue endothelial dysfunction. *Hypertension*.

[41] Verdonk K., Saleh L., Lankhorst S. (2015). Association studies suggest a key role for endothelin-1 in the pathogenesis of preeclampsia and the accompanying renin–angiotensin–aldosterone system suppression. *Hypertension*.

[42] Gregory A. L., Xu G., Sotov V., Letarte M. (2014). The enigmatic role of endoglin in the placenta. *Placenta*.

[43] Ahmed A. (2011). New insights into the etiology of preeclampsia: identification of key elusive factors for the vascular complications. *Thrombosis Research*.

[44] Hashimoto Y., Ishida J., Yamamoto R. (2005). G protein-coupled APJ receptor signaling induces focal adhesion formation and cell motility. *International Journal of Molecular Medicine*.

[45] Zeng H., He X., Hou X., Li L., Chen J. X. (2014). Apelin gene therapy increases myocardial vascular density and ameliorates diabetic cardiomyopathy via upregulation of sirtuin 3. *American Journal of Physiology - Heart and Circulatory Physiology*.

[46] Tenório M. B., Ferreira R. C., Moura F. A., Bueno N. B., de Oliveira A. C. M., Goulart M. O. F. (2019). Cross-talk between oxidative stress and inflammation in preeclampsia. *Oxidative Medicine and Cellular Longevity*.

[47] Chiarello D. I., Abad C., Rojas D. (2020). Oxidative stress: normal pregnancy versus preeclampsia. *Biochimica et Biophysica Acta (BBA)-Molecular Basis of Disease*.

[48] Murthi P., Pinar A. A., Dimitriadis E., Samuel C. S. (2020). Inflammasomes—a molecular link for altered immunoregulation and inflammation mediated vascular dysfunction in preeclampsia. *International Journal of Molecular Sciences*.

[49] Armistead B., Kadam L., Drewlo S., Kohan-Ghadr H.-R. (2020). The role of NF*κ*B in healthy and preeclamptic placenta: trophoblasts in the spotlight. *International Journal of Molecular Sciences*.

[50] Pisarenko O., Shulzhenko V., Studneva I. (2015). Structural apelin analogues: mitochondrial ROS inhibition and cardiometabolic protection in myocardial ischaemia reperfusion injury. *British Journal of Pharmacology*.

[51] Duan J., Cui J., Yang Z. (2019). Neuroprotective effect of Apelin 13 on ischemic stroke by activating AMPK/GSK-3*β*/Nrf2 signaling. *Journal of Neuroinflammation*.

[52] Tian Y., Chen R., Jiang Y., Bai B., Yang T., Liu H. (2020). The protective effects and mechanisms of apelin/APJ system on ischemic stroke: a promising therapeutic target. *Frontiers in Neurology*.

[53] Raafat N. A., Fathy M. A. (2018). Serum adropin levels in a preeclampsia like L-name rat model treated with sildenafil citrate. *The Medical Journal of Cairo University*.

[54] Tal R., Shaish A., Barshack I. (2010). Effects of hypoxia-inducible factor-1*α* overexpression in pregnant mice: possible implications for preeclampsia and intrauterine growth restriction. *American Journal of Pathology*.

[55] Hatice A., Suleyman A., Zeki I. A. (2015). Adipocytokines in particular pregnancy disorders. *Annals of Clinical and Laboratory Research*.

