# Supplementary material for: Correlation between Apelin and Some Angiogenic Factors in the Pathogenesis of Preeclampsia: Apelin-13 as Novel Drug for Treating Preeclampsia and Its Physiological Effects on Placenta
Source: Int J Endocrinol. 2021 Nov 15;2021:5017362. doi: 10.1155/2021/5017362 (PMC8608536; doi:10.1155/2021/5017362)
Supplement: Supplementary Materials — Attached supplementary files show measurement of the blood pressure of animals and anatomical features of pregnant rats. [file 5017362.f1.doc]

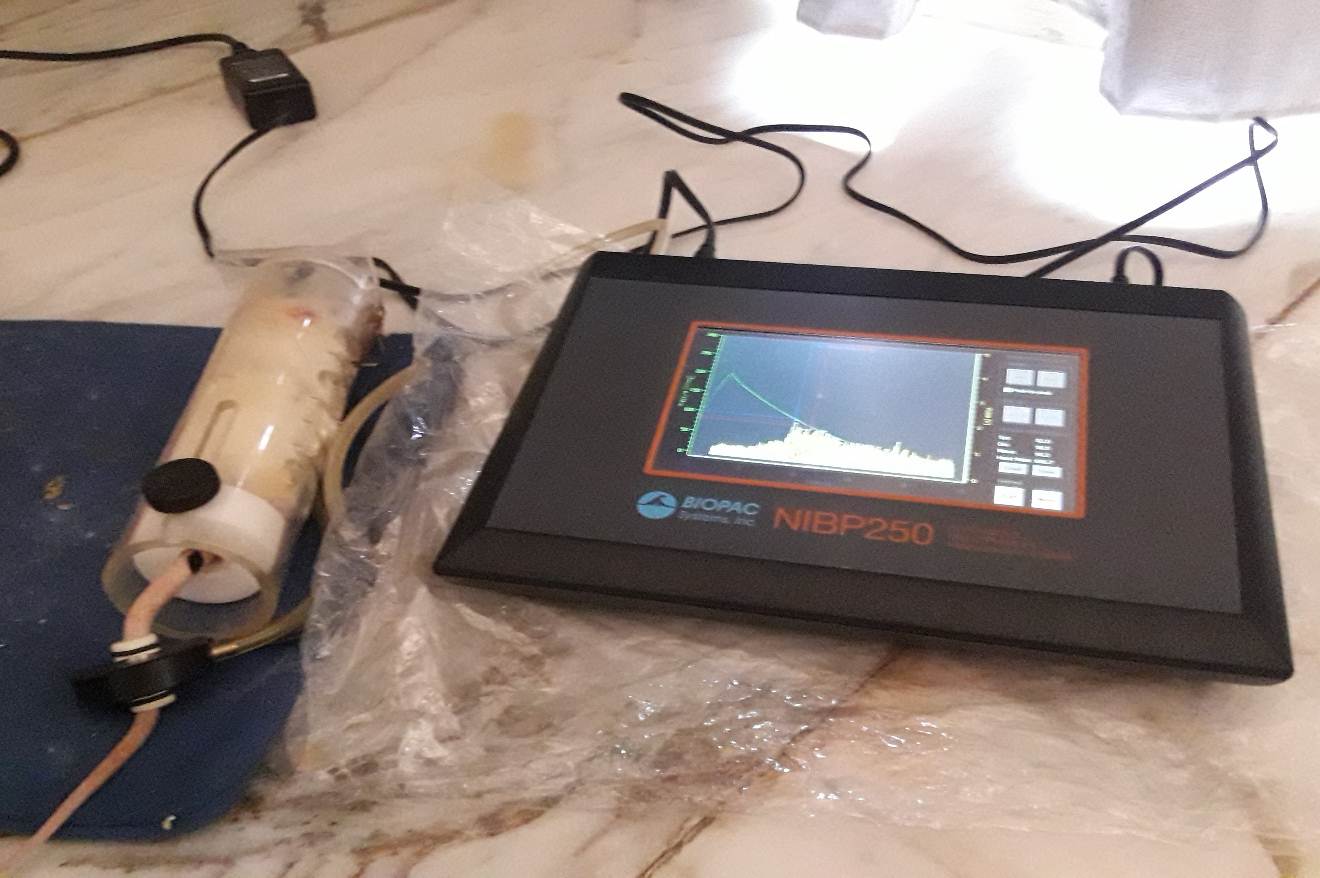

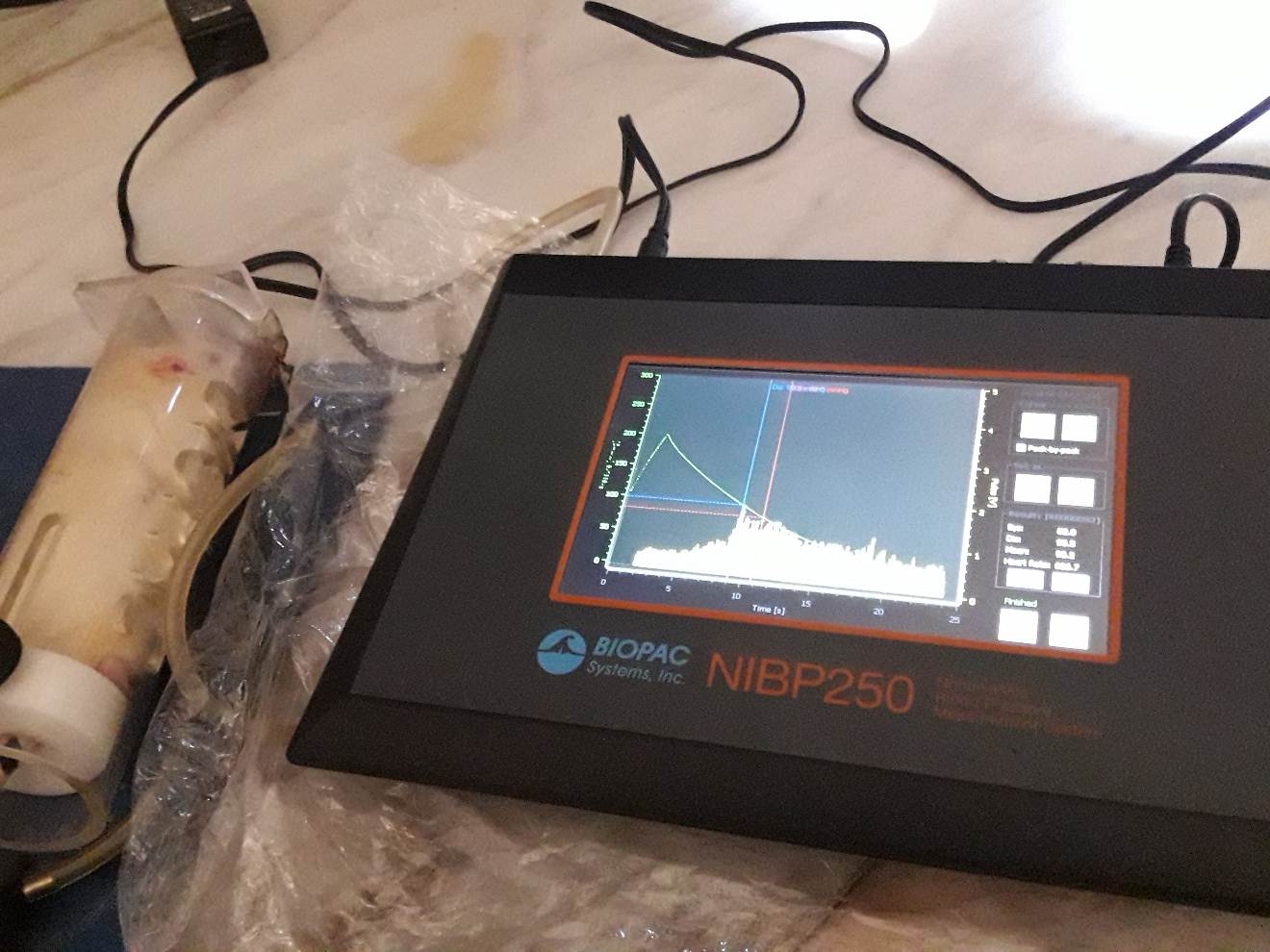
**Blood Preseeure measurment by NIBP250 (BIOPAC)**


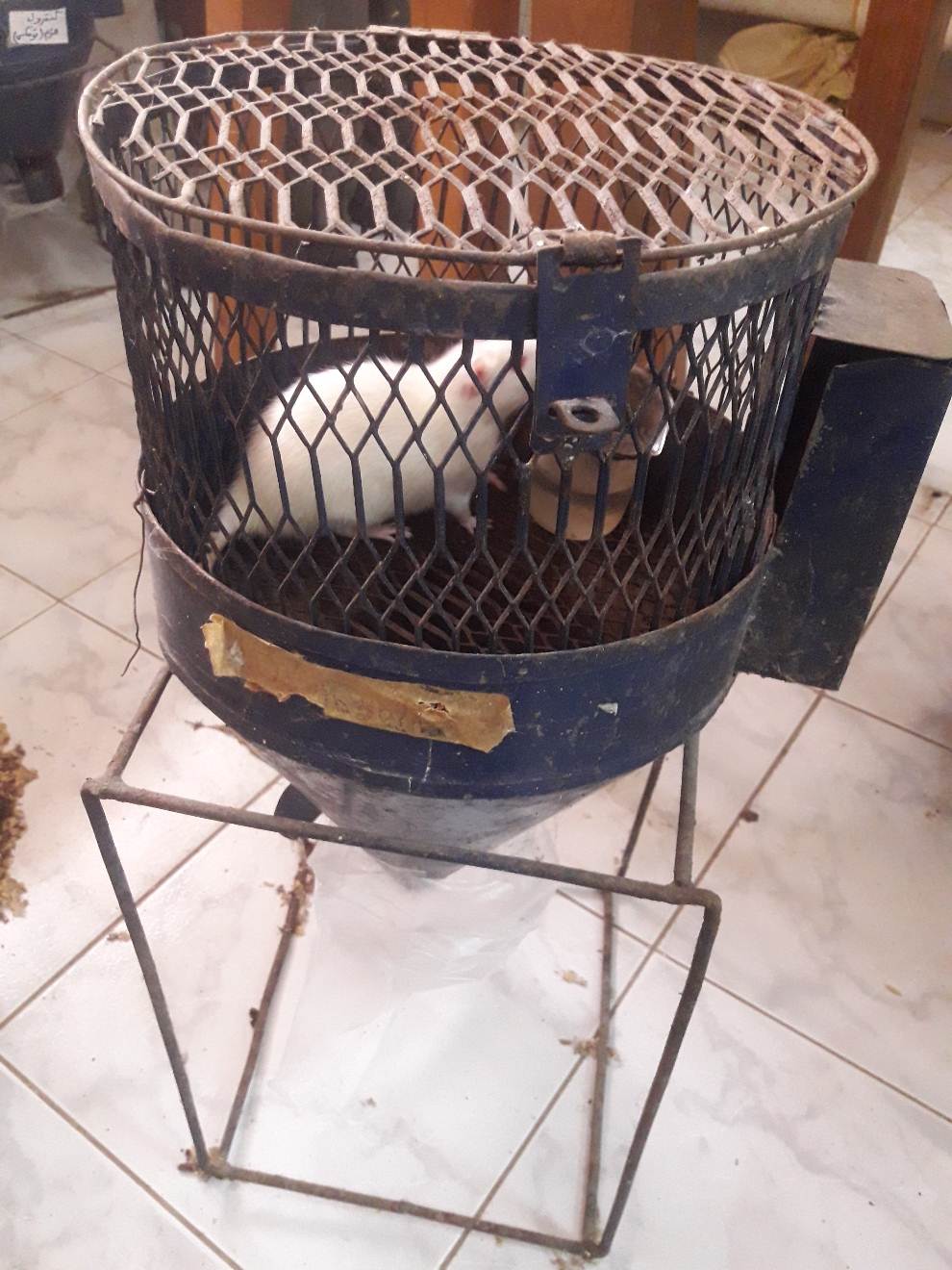


**Urine Samples collection**


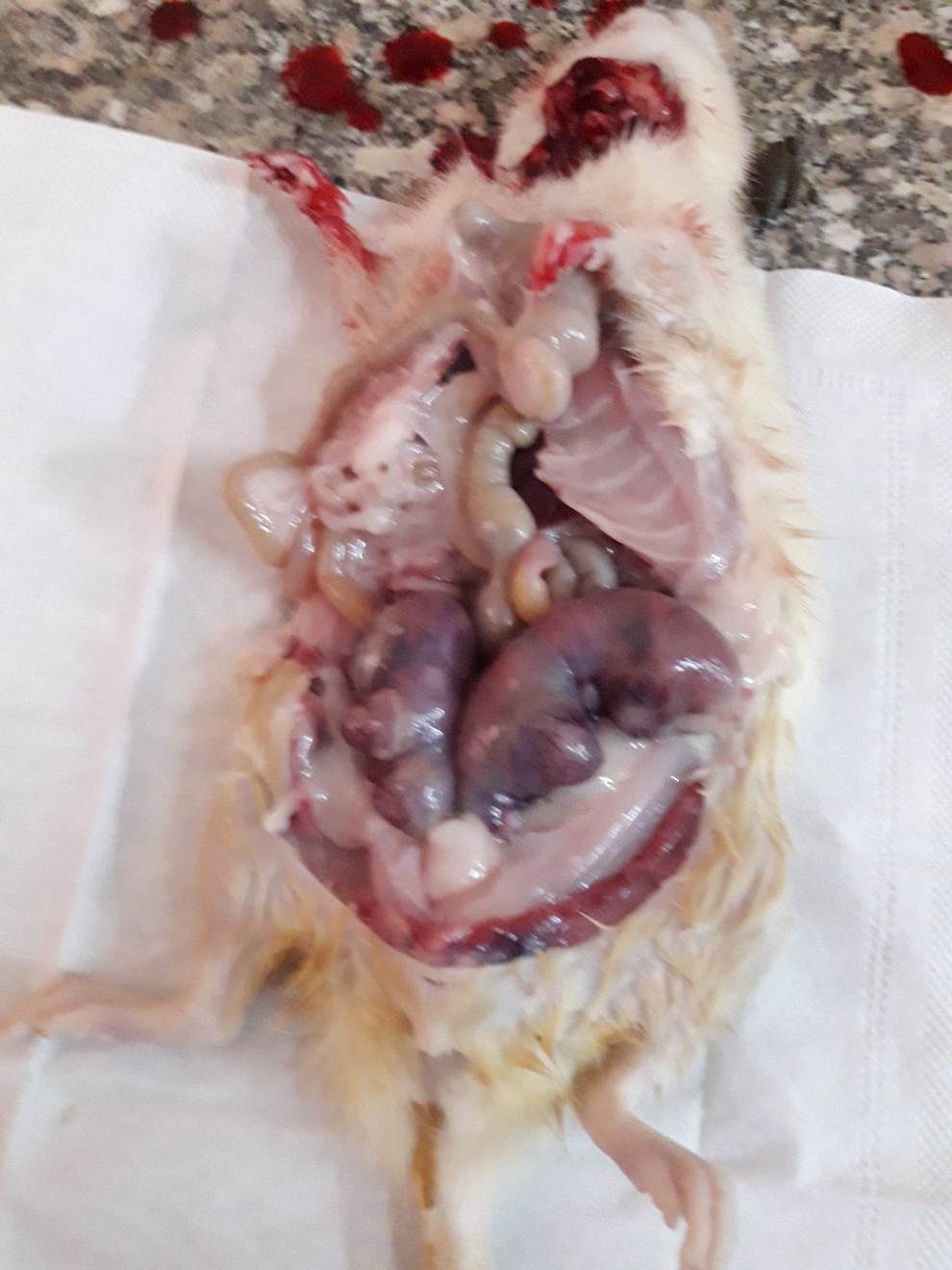

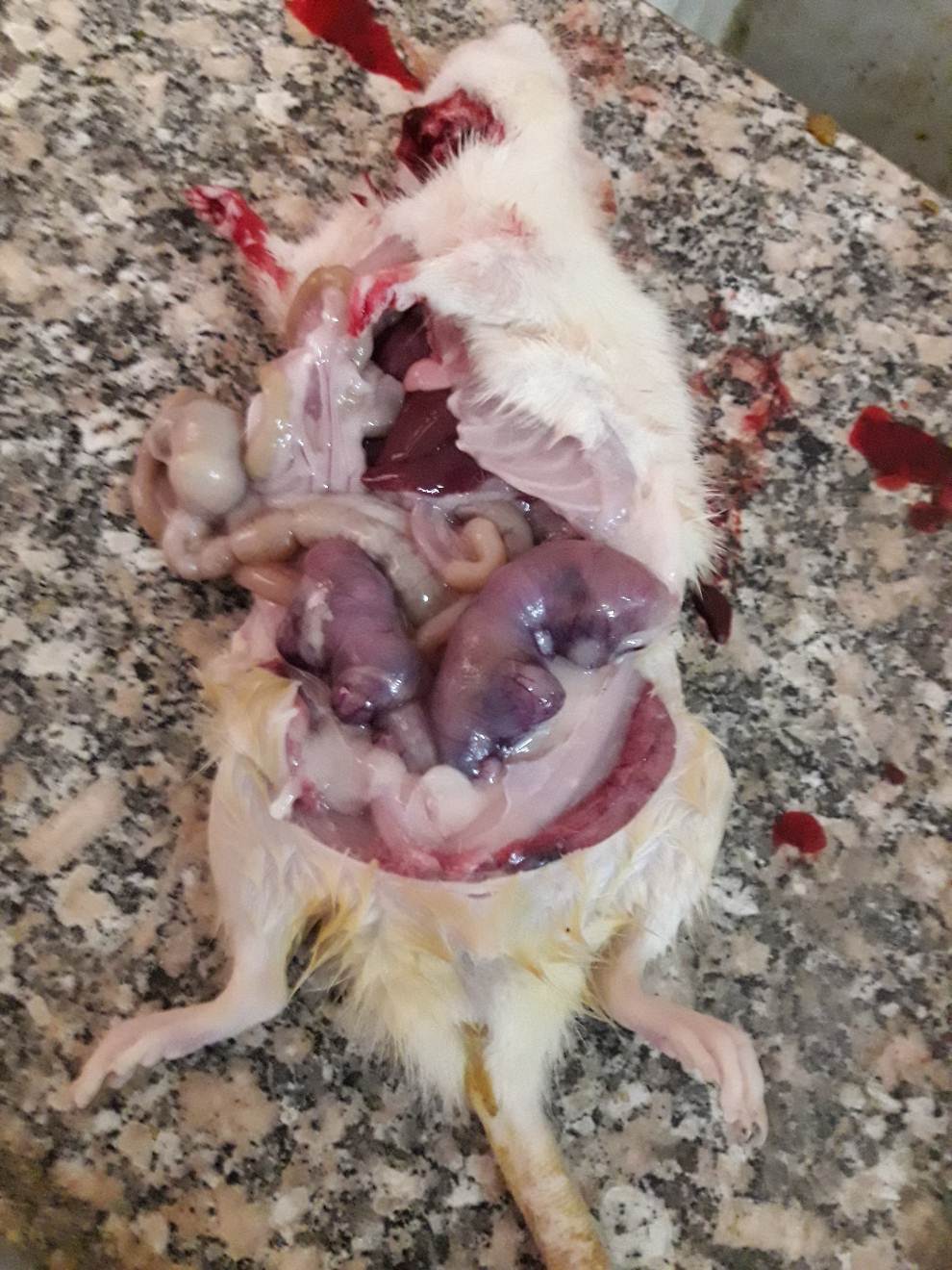

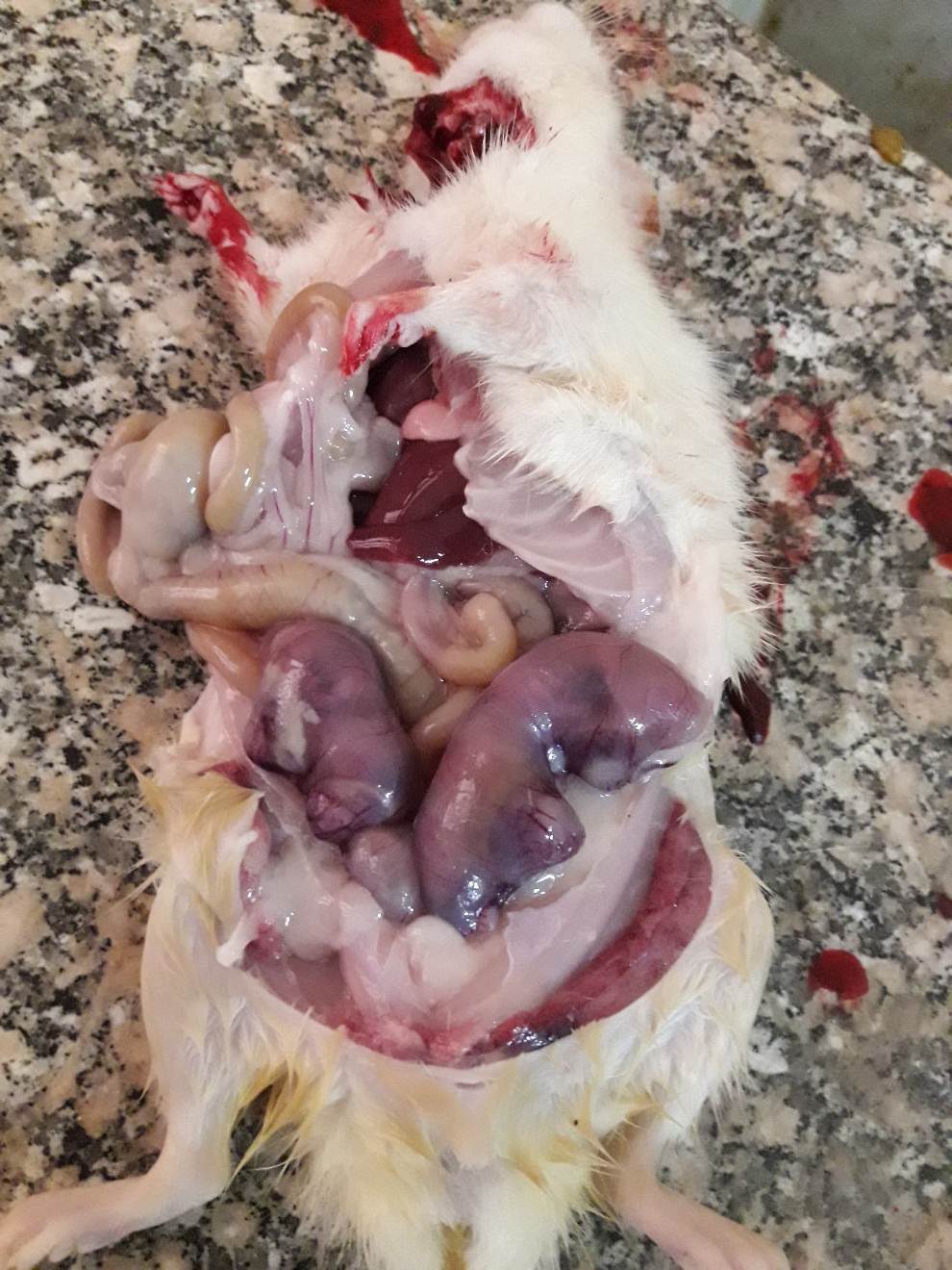


**Pregnant rats from different treated groups**
